# Dysfunction of the **Mesolimbic Circuit** to **Food Odors** in **Women With** Anorexia and Bulimia Nervosa: A fMRI **Study**

**DOI:** 10.3389/fnhum.2019.00117

**Published:** 2019-04-04

**Authors:** Tao Jiang, Robert Soussignan, Edouard Carrier, Jean-Pierre Royet

**Affiliations:** ^1^Olfaction: From Coding to Memory Team, Lyon Neuroscience Research Center, CNRS UMR 5292, INSERM U1028, UCBL, Centre Hospitalier Le Vinatier, Bron, France; ^2^Developmental Ethology and Cognitive Psychology Group, Centre des Sciences du Goût et de l’Alimentation, CNRS (UMR 6265), Université de Bourgogne-Inra, Dijon, France; ^3^Clinique Saint Vincent de Paul, Lyon, France

**Keywords:** eating disorders, reward circuit, liking, wanting, energy-density food, metabolic state, olfaction

## Abstract

Brain reward dysfunction in eating disorders has been widely reported. However, whether the neural correlates of hedonic and motivational experiences related to food cues are differentially affected in anorexia nervosa of restrictive type (ANr), bulimia nervosa (BN), and healthy control (HC) participants remains unknown. Here, 39 women (14 ANr, 13 BN, and 12 HC) underwent fMRI while smelling food or non-food odors in hunger and satiety states during liking and wanting tasks. ANr and BN patients reported less desire to eat odor-cued food and odor-cued high energy-density food (EDF), respectively. ANr patients exhibited lower ventral tegmental area (VTA) activation than BN patients to food odors when rating their desire to eat, suggesting altered incentive salience attribution to food odors. Compared with HC participants, BN patients exhibited decreased activation of the caudate nucleus to food odors in the hunger state during the wanting task. Both patient groups also showed reduced activation of the anterior ventral pallidum and insula in response to high EDF odors in the hunger state during the wanting task. These findings indicate that brain activation within the food reward-regulating circuit differentiates the three groups. ANr patients further exhibited lower activation of the precuneus than other participants, suggesting a possible role of body image distortion in ANr. Our study highlights that food odors are relevant sensory probes to gain better insight into the dysfunction of the mesolimbic and striatal circuitry involved in food reward processing in patients with EDs.

## Introduction

Anorexia nervosa (AN) and bulimia nervosa (BN) are severe, multi-faceted eating disorders (EDs) of unknown etiology involving biological, psychological and sociocultural factors (Connan et al., 2003; Kaye et al., 2013; Monteleone et al., 2017). AN is characterized by a persistent restriction of energy intake, leading to significantly decreased body weight, an intense fear of gaining weight, and disturbed perception of one’s body weight or shape; BN is characterized by recurrent episodes of binge eating associated with inappropriate/unhealthy compensatory behaviors (e.g., purging) to avoid weight gain (American Psychiatric Association, 2013). These EDs share some personality traits, such as anxiety, obsessive-compulsiveness, and harm avoidance, but they consistently differ in inhibitory self-control (Anderluh et al., 2003; Kaye et al., 2004). AN patients are overcontrolled, whereas BN patients are more impulsive and sensation/novelty seeking. Furthermore, AN patients are less sensitive to reward than healthy control (HC) participants and experience less pleasure from the anticipation of palatable foods than HC or BN participants (Harrison et al., 2010; Soussignan et al., 2011).

Neuroimaging research on the hemodynamic correlates of the processing of disorder-related (food cues/intake, body image perception) and disorder-unrelated (monetary gain) stimuli in chronically ill or recovered women with AN and BN has consistently reported altered brain functions in the fronto-striatal and limbic regions (Friederich et al., 2013; Kaye et al., 2013; Monteleone et al., 2017). Thus, aberrant processing in brain regions underlying reward value coding and reward-based decision making, including the striatum, anterior insula, anterior cingulate cortex, orbitofrontal cortex (OFC), and ventral tegmental area (VTA), have been involved in the pathophysiology of EDs (Berridge, 2009; Broft et al., 2011; Weygandt et al., 2012; Park et al., 2014). However, the interpretation of results remains unclear, as inconsistent findings have been reported. For instance, AN patients revealed increased responses in brain reward regions (e.g., ventral or dorsal striatum, insula, OFC) to the sight and/or taste of food compared with HC, which suggest that they may have attributed increased salience to food stimuli (Uher et al., 2004; Gizewski et al., 2010; Cowdrey et al., 2011; Frank et al., 2012; Oberndorfer T. et al., 2013; Sanders et al., 2015). Hypo-responsivity to the sight of food images (Holsen et al., 2012) or taste stimuli (Wagner et al., 2008) in regions of brain reward has also been evidenced in such patients, suggesting that they might be less prone to process the hedonic aspects of food. These inconsistent findings could be related to differences between sample characteristics (recovery or acute illness), motivational factors (e.g., fasting period), and to the nature of task-dependent reward processing (e.g., passive observation or explicit reward instruction). Indeed, it has been emphasized that active engagement (vs. passive observation) in a reward paradigm is needed to appropriately capture the nature of reward processing (Katsyri et al., 2013).

In the classical taste-reactivity paradigm, food reward is mainly operationalized by a motivational component termed “wanting” (attribution of incentive salience to a reward and effort to obtain it) and by an affective component termed “liking” (hedonic impact of the reward) (Berridge, 2009). Food odors are potent stimuli that induce visceral conditioned cues and anticipatory signals for ingestion (Jansen, 1998; Soussignan et al., 2012). We recently investigated the subjective experience of food liking and wanting evoked by odors in HC participants and observed that wanting and liking tasks induced segregated striatal activation (Jiang et al., 2015). Here, we investigated whether a pattern of differential activation in mesolimbic circuitry could distinguish AN of restrictive type (ANr) and BN women from HC participants. We explored whether the participants in the three groups are differentially characterized by either elevated or reduced sensitivity in reward-processing brain areas in response to food odors while successively performing liking and wanting tasks in hungry and satiated states. These tasks have previously been used to evidence aberrant behavioral responses to food images in ANr (Cowdrey et al., 2013) and in a normal population with a greater binge-eating trait (Finlayson et al., 2011), but not, to our knowledge, in a neuroimaging study comparing the respective neural correlates of affective and motivational components of reward-related behaviors to food odors in ANr and BN.

## Materials and Methods

### Participants

Twelve HC participants, 14 ANr patients and 13 BN patients, matched in age, participated in the study. All participants were right-handed women, handedness being checked by the Edinburgh Handedness Inventory (Oldfield, 1971). HC participants were recruited from the university community with newspaper advertisements and fliers. The patients were recruited from the Clinique Saint Vincent de Paul (Lyon, France). Diagnostic assessment was based on the Structured Clinical Interview for the DSM-IV Axis I disorders (American Psychiatric Association, 1994). All patients met the criteria for a principal diagnosis of ANr or BN. The ANr patients were hospitalized because of very low body mass index (BMI) and malnutrition related to severe body weight loss. The BN patients were characterized by purging and binge-eating behaviors. Participants were screened for the presence or absence of EDs using the Bulimic Inventory Test, Edinburgh (BITE) (Henderson and Freeman, 1987). ANr and BN patients had significantly higher scores than HC participants (*p* < 0.001), and BN patients had significantly higher scores than ANr patients (*p* = 0.007). Participants further completed several self-report assessments (Table 1). Because high rates of obsessive-compulsive and depressive symptoms (Kennedy et al., 1994), alexithymia (i.e., difficulties identifying and describing one’s own emotions) (Lule et al., 2014), disgust sensitivity (Aharoni and Hertz, 2012), and anhedonia (Raffi et al., 2000) have been previously reported in AN and/or BN patients, we assessed these psychopathological traits using the Beck Depression Inventory (BDI) (Beck and Beamesderfer, 1974), the difficulty describing feelings subscale of the Toronto Alexithymia scale (TAS) (Bagby et al., 1994), the Disgust Propensity and Sensitivity Scale (DPSS) (Van Overveld et al., 2006), and the physical anhedonia scale (Chapman et al., 1976).

**Table 1:** Descriptive statistics of demographic, clinical, and physiological parameters.

	HC	ANr	BN	*F*	*df*	*P*
**Demographic variables**					
N	12	14	13			
Age (years)	24.14 ± 3.06	24.94 ± 4.67	22.50 ± 2.88	1.524	2.36	0.232
**Clinical variables**					
BMI	21.45 ± 2.66	15.83 ± 0.97	20.42 ± 4.20	14.25	2.36	<0.001
BITE	4.00 ± 3.22	18.00 ± 7.28	23.93 ± 4.56	44.46	2.36	<0.001
BDI	1.09 ± 1.38	13.62 ± 4.79	16.58 ± 7.63	24.28	2.37	<0.001
TAS	12.13 ± 3.23	24.83 ± 4.63	24.30 ± 6.90	15.21	2.30	<0.001
DPSS	73.67 ± 11.93	92.85 ± 18.85	81.92 ± 22.98	6. 39	2.37	0.040
Anhedonia	10.42 ± 5.84	16.46 ± 9.40	18.58 ± 4.46	9.12	2.37	0.010
**Odor detection and breathing cycle**					
Odor detection	84.7 ± 15.1	93.6 ± 11.3	98.2 ± 2.9	5.106	2.36	0.011
Breathing cycle	4.00 ± 0.85	4.33 ± 1.42	4.25 ± 1.11	0.281	2.36	0.756

*ANr, anorexia nervosa of restrictive type; BN, bulimia nervosa; HC, healthy control; BMI, body mass index; BITE, Bulimic Inventory Test, Edinburgh; BDI, Beck Depression Inventory; DPSS, Disgust Propensity and Sensitivity Scale. TAS, Toronto Alexithymia scale. The mean and standard deviation are given for each parameter and group. F, F-test statistic; df, degrees of freedom; p, probability.*

Exclusion criteria for all groups were major medical illness, current antidepressant or other psychiatric medication, lifetime history of attention deficit hyperactivity disorder, anxiety disorder, current or past schizophrenia, bipolar disorder, and alcohol or substance abuse. All the participants were further without known olfactory impairments, rhinal disorders (colds, active allergies, a history of nasal-sinus surgery, or asthma), pregnancy, neurological diseases, ferrous implants (e.g., pacemakers and cochlear implants), food allergies, or claustrophobia. They were selected after screening for their olfactory detection ability with a forced-choice suprathreshold detection test (at least 85% of correct responses) and the mean duration of their breathing cycle (from 4.00 to 6 s/cycle). We applied this breath cycle criteria to optimize the efficiency of olfactory stimulation.

The study was conducted in accordance with the Declaration of Helsinki. Participants were informed about the procedure of the tasks, and provided informed written consent as required by the local Institutional Review Board according to French regulations on biomedical experiments with healthy volunteers (Ethical Research Committee of CPP-Sud Est II (n° CPP A07-03), DGS2007-0054, February 23, 2007). Each participant received 150 euros for participation. Handedness was checked with the Edinburgh Handedness Inventory (Oldfield, 1971).

### Stimuli

The detailed methodology has been previously reported (Jiang et al., 2015). Briefly, 56 odorants were used: 28 for training purposes and 28 for the fMRI scanning session. For fMRI, odorants were composed of 14 food odorants and 14 Nfood odorants not evoking food (Table 2). They were diluted in odorless mineral oil (Sigma Aldrich, Saint-Quentin-Fallavier, France) to different concentrations in volume to equalize their subjective intensity (Jiang et al., 2015). For stimuli presentation, 5 ml of solution was absorbed into compressed polypropylene filaments inside a 100 ml white polyethylene squeeze-bottle equipped with a dropper (Fisher Scientific, Illkirch, France).

**Table 2:** List of odorants selected to evoke food and Nfood.

Food			Nfood		
Number	Label	Conc. (%)	Number	Label	Conc. (%)
1	Apricot	5.0	1	Camphor	10.0
2	Bacon	5.0	2	Carnation	10.0
3	Banana	20.0	3	Citronella	20.0
4	Beef	10.0	4	Cleaning product	20.0
5	Bitter almond	5.0	5	Fresh grass	5.0
6	Blue cheese	2.5	6	Hyacinth	10.0
7	Gruyère cheese	2.5	7	Lavender	20.0
8	Pâté	1.0	8	Pine	10.0
9	Peanut	5.0	9	Rose	10.0
10	Pizza	1.0	10	Soap	5.0
11	Potato	0.5	11	Tar	10.0
12	Shellfish	3.0	12	Tobacco	10.0
13	Smoked bacon	2.0	13	Violet	10.0
14	Strawberry	10.0	14	White spirit	10.0

*Nfood, non food; Conc, concentration.*

### Stimulating and Recording Materials

They were presented to the participants using an airflow olfactometer, which allows the stimuli to be synchronized with breathing (Vigouroux et al., 2005). Participants’ responses were acquired with a five key-press button box that provided logic signals. The five buttons were placed in a configuration similar to the five fingers (thumb, forefinger, middle finger, ring finger, and pinkie) of the right hand, corresponding to the five levels of a visual rating scale. Breathing was recorded using polyvinyl-chloride foot bellows (Herga Electric Limited, Suffolk, United Kingdom) secured to the subject’s abdomen with a cotton belt. Participants’ behavioral responses, breathing data, stimulation onset, and trigger signals from the MRI scanner were recorded online (100 Hz sampling rate) using a laptop equipped with a digital acquisition board I/O card (PCI-6527) (National Instruments^®^, Austin, TX, United States) using the LabVIEW software package (National Instruments^®^). Data were further analyzed using custom routines created with MATLAB (The MathWorks, Natick, MA, United States).

### Experimental Procedure

**FIGURE 1:**
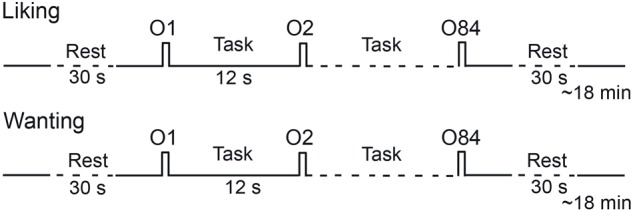
Timeline of the procedure for a session. Each session performed in the morning (hunger state) or the afternoon (satiety state) included two runs during which liking and wanting tasks were performed, respectively. O, odor.

Two sessions were planned for each participant on two consecutive days. Participants were alternatively scanned in hunger and satiety states. In each session, two functional runs were performed during which the subjects, respectively, reported their odor liking and wanting (Figure 1). During each run, 28 odorants were delivered three times each, such that 84 stimuli were presented. They were delivered according to an event-related fMRI design with a jittered interstimulus interval of ∼12 s, depending on the participant’s respiration. The orders of the two sessions (hunger vs. satiety) and runs (liking vs. wanting) were counterbalanced across participants. However, the same run order was performed between two metabolic states for a given participant. In other words, a participant rated in the hunger state first with the liking task then second with the wanting task, was also tested in the same order in the satiated state. The order of the presentation of odorants was randomized for each run.

During the liking run, the participants were asked to press one of five buttons using the corresponding finger, depending on their liking judgment (thumb: very unpleasant; forefinger: unpleasant; middle finger: neutral; ring finger: pleasant; pinkie: very pleasant). During the wanting run, if the odor evoked food, the participants were asked to press one of five buttons, depending on their desire to eat the food evoked by the odor (not at all, not desired, just a little, much desired, urge). If the odor did not evoke food, they did not press a button.

For the fasted (hunger) state, participants were scanned at either 11:30 a.m. or 0:30 p.m. and reported that they complied with the requirement to take their breakfast (only tea or coffee, plus a slice of bread) not later than 7:00 or 8:00 a.m, respectively. For the satiated state, subjects were scanned at either at 1:30 or 2:30 p.m. Four participants were tested within a day, two in the morning in the hungry state (11:30 a.m. and 0:30 p.m.), 4–5 h following their daily breakfast, and two in the afternoon in the satiated state (1:30 and 2:30 p.m.), 30 min after having taken a standard meal at the Imaging Center. The meal was composed of salad (150 g), a filet of beef (150 g), green beans (150 g), Gruyère cheese (30 g), and plain yoghurt (125 g). The participants’ hunger/satiety states were evaluated at the onset and end (pre-meal and post-meal) of each fMRI session using a 10-point Likert-type scale (1 = no hunger at all; 10 = extremely hungry).

### Behavioral Data Analysis

Because liking and wanting scores are correlated (Jiang et al., 2008, 2013), we performed multivariate analysis of variance (MANOVA) using task (liking vs. wanting) and state (hunger vs. satiety) factors with repeated measurements on the odorant factor (Winer et al., 1991). Analysis of variance (ANOVA) with repeated measurements was then used to separately analyze the scores derived from the judgment tasks. The differences between pairs or groups of means were assessed using multiple orthogonal contrasts.

As breathing variations are known to impact brain activation (Sobel et al., 1998), the amplitudes of the inspiratory and expiratory waveforms and the amplitudes of the inspiratory cycle following each odor stimulation and the cycle preceding the stimulation were estimated. The performance of the participants in identifying the odor category (food vs. Nfood) during the wanting task was also assessed.

Identification performance of odor category (food vs. Nfood) during the wanting task was assessed using the parameters of signal-detection theory (Banks, 1970; Lockhart and Murdock, 1970). By combining the experimental condition (food or Nfood) and the participant’s behavioral response (correct or incorrect), four outcomes were scored. If during the wanting task the stimulus was a food odor and the participant pressed one of five buttons, a “hit” was scored. If the participant did not press one of five buttons, a “miss” was scored. If the stimulus was a Nfood odor and the participant did not press one of five buttons, a “correct rejection” was scored. If the participant incorrectly pressed one of five buttons, a “false alarm” (FA) was scored. From the hit and FA scores, four parameters were then calculated for each participant: the hit rate (*HR*), FA rate (*FR*), discrimination measurement *d’_L_*, and bias response (*C_L_*). Corwin (1989) previously described these calculations in the framework of different paradigms of yes-no tasks as follows:

(1) $${d^{\prime }}_{L}=ln\frac{HR(1-FR)}{FR(1-HR)}$$

(2) $$C_{L}=0.5\times ln\frac{\left(1-FR)(1-HR\right)}{HR\times FR}$$

where *HR* represents the hit rate [(Hit + 0.5)/(*N*_1_ + 1)], *FR* represents the FA rate [(*FA* + 0.5)/(*N*_2_ + 1)], and *N*_1_ and *N*_2_ represent the number of trials with food odor and Nfood odor, respectively, for which the participants provided an answer. The discrimination (*d’_L_*) score may be good or poor (positive and negative values, respectively). The response bias (*C_L_*) scores establish three individual attitudes. The participants may be conservative (tending to respond “no” to a food odor), neutral (responding “yes” or “no” with equal probability) or liberal (tending to respond “yes”), denoted by positive, neutral or negative values of *C_L_*, respectively (Snodgrass and Corwin, 1988). One-way ANOVA were used to compare between-group performances.

Behavioral and breathing data were analyzed using open source Python scripts. To control for the Type I error rate associated to multiple comparisons, we applied the Bonferroni correction by dividing the probability alpha by the number of comparisons. Statistical analyses were performed using Statistica (StatSoft^®^, Tulsa, OK, United States).

### Functional Data Analysis

Images were acquired using a 1.5-Tesla MAGNETOM Sonata whole-body imager (Siemens Medical^®^, Erlangen, Germany) equipped with a 4-channel circularly polarized head coil. For each functional imaging scan, we obtained 26 interleaved, 4 mm-thick axial slices using a T2^∗^-weighted echo-planar sequence. In total, 460 scans were collected for each functional run. A high-resolution structural T1-weighted anatomical image (inversion-recovery 3D Gradient-Echo sequence, 1 × 1 × 1 mm) parallel to the bicommissural plane and covering the entire brain was acquired over ∼10 min.

We processed all functional images using Statistical Parametric Mapping software (SPM5, Wellcome Department of Cognitive Neurology, London, United Kingdom) (Friston et al., 1995a). All functional volumes were slice-timing corrected, realigned to the median volume, coregistered to the anatomical image, spatially normalized to the Montréal Neurological Institute (MNI) standard brain (Friston et al., 1995b), and smoothed with a 7 × 7 × 8 mm full-width half-maximum Gaussian kernel, which is considered to be optimal for both single-subject inference and for group inference in statistical parametric maps (Mikl et al., 2008). Preprocessed data were statistically analyzed on a subject-by-subject basis using the general linear model. The regressors were modeled by convolving a neural model derived from the stimuli onsets with a hemodynamic response function (hrf).

For each subject, activation associated with six conditions of interest [category (food, Nfood), state (hunger, satiety), and task (liking, wanting)] was modeled using boxcar predictors convolved with both the canonical hrf and its time derivative (Friston et al., 1998; Hopfinger et al., 2000). A high-pass filter (cut-off frequency of 1/120 Hz) was used to eliminate instrumental and physiological signal fluctuations at very low frequencies. As the hrf varies depending on the subject and area of interest (Handwerker et al., 2004), we attempted to better estimate this function using both the canonical hrf and its time derivative (Hopfinger et al., 2000). Because of uncertainty in the onset of sniffing the odor, we used the amplitude of the hrf in group random-effect analysis, which removes potential bias in results caused by latency (Kühn and Gallinat, 2012). Stimulus onset asynchronies were fixed at the time of odor delivery. Random-effects analyses were performed to extrapolate statistical inferences at the population level, as described in the SPM5 software.

Analyses were first performed on brain regions of interest (VOI, volume of interest) known to be involved in food reward processing (Mawlawi et al., 2001; Liu et al., 2011; Diekhof et al., 2012; Berridge and Robinson, 2016). These VOIs were the ventral and dorsal striatopallidum, two areas of the midbrain (VTA and substantia nigra), the anterior part of the insula and the medial OFC for which we previously evidenced that activation in this area was positively correlated with the wanting scores in the hunger state (Jiang et al., 2015). To improve relevance of findings, comparisons between activation data were performed only when differences between behavioral performances were significant. For wanting, comparisons were further performed for the food condition only, as participants provided responses only for odors evoking food.

VOIs were drawn from the MNI template (Ch2better.nii) using MRIcron^1^ and human brain atlases (Duvernoy, 1999; Mai et al., 2008). The ventral and dorsal striatopallidum was drawn from coronal slices (positive and negative MNI y values indicate anterior and posterior coordinates to the anterior commissure, respectively). It was subdivided into five sub-regions that were the nucleus accumbens (NAc) (from *y* = 19 to 5 mm anterior to the anterior commissure), the anterior ventral pallidum (VP) (from 5 to 3 mm), the caudate nucleus (from 25 to -8 mm), the globus pallidus (from 3 to -10 mm), and the putamen (from 21 to -21 mm). The VTA and the substantia nigra were drawn from -8 to -10 mm and from -8 to -21 mm, respectively. For these regions, delineations were based on the visual differentiation of structures (T1 images) and on the detailed diagrams and pictures from the Mai et al. (2008)’s atlas. The medial orbital gyrus was drawn from coronal slices medial (from 56 to 6 mm), using the Duvernoy (1999)’s atlas. The anterior insula was drawn from 5 to -31 mm and delineations were mainly based on coronal and axial sections of the Duvernoy (1999)’s atlas. Three-ways (side × state × group) ANOVAs were performed for each VOI data. We considered only the pair-wise comparisons resulting from significant main effects or interactions taking into account the group factor (ANr/BN, ANr/HC, and BN/HC). To control for the Type I error rate associated to multiple comparisons, we performed non parametric permutation tests (10,000 permutations) in order to determine adjusted *p*-values from the distribution of the T_max_ statistic (e.g., Nichols and Holmes, 2001; Chen et al., 2013). For each permutation test, the T_max_ was the maximal *t*-value obtained by considering all VOIs and all contrasts (e.g., ANr/BN, ANr/HC, and BN/HC).

Second, as an exploratory study, whole-brain analyses were performed on functional images for the different experimental conditions. We compared activation functional images among the three groups ([HC vs. ANr], [HC vs. BN], and [ANr vs. BN]) as a function of four conditions of interest (food/Nfood, hunger/satiety) for the two rating tasks ([liking – baseline] and [wanting – baseline] contrasts). The level of significance was set at *P* = 0.001, uncorrected at the cluster level for multiple comparisons across the much larger volume of the whole brain. We reported significant activations at a threshold of *P* = 0.005 uncorrected in regions predicted *a priori*, such as the caudate nucleus. We used an extent threshold (k) superior or equal to 6 adjacent activated voxels to reduce the observation of false-positive events, but by keeping the chance to observe activation in small structures as the piriform cortex or areas of the striatopallidum. As for VOI analyses, comparisons between activation functional images were applied only when differences between behavioral performances were significant.

## Results

### Behavioral and Physiological Data

#### Demographic and Behavioral Characteristics

The participants’ demographic and behavioral characteristics are summarized in Table 1. The BMI differed significantly among the three groups of subjects and was found to be lower in ANr than in HC or BN participants (*P*’s < 0.001). Furthermore, ANr and BN patients were more depressed (*P*’s < 0.001) and had more trouble identifying emotions (*P*’s ≤ 0.002) than HC participants. ANr patients reported more disgust than HC participants (*P* = 0.035), and BN patients presented more anhedonia than HC participants (*P* = 0.009). In olfactory detection, suprathreshold detection scores differed among the three groups, with higher detection scores in ANr and BN than HC participants (*P* = 0.038 and *P* = 0.003, respectively).

#### Metabolic State

**FIGURE 2:**
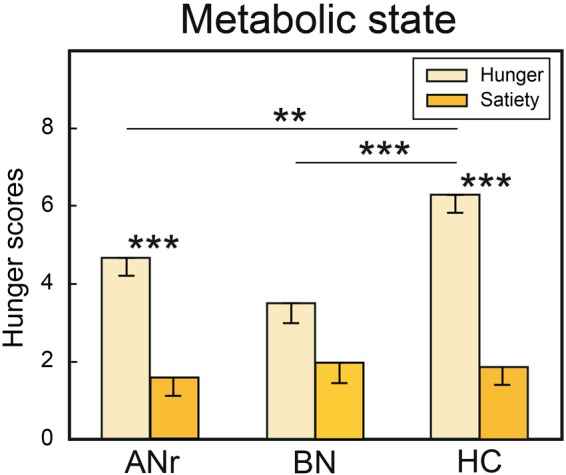
Hunger scores when the subjects of the three groups were tested in hunger and satiety states. HC, healthy control; ANr, anorexia nervosa of restrictive type; BN, bulimia nervosa. Vertical bars, standard errors of the mean (s.e.m.); ^∗∗^*P* < 0.01; ^∗∗∗^*P* < 0.001.

The hunger scores collected at the onset and end of each fMRI session (pre-meal and post-meal) revealed a significant state × group interaction [*F*(2, 36) = 6.051, *P* = 0.005], indicating that HC and ANr participants (Figure 2), but not BN participants (*P* = 0.012, α_adjusted_ = 0.008), scored higher when they were tested in the hunger state than in the satiety state (*P’s* < 0.001). In the hunger state, the scores were also higher in HC participants than in ANr (*P* = 0.002) and BN (*P* < 0.001) patients, but not significantly higher in ANr patients than in BN patients (*P* = 0.024, α_adjusted_ = 0.008). These data demonstrated that participants in the three groups did not report the same hunger state. We further observed a significant time effect [*F*(1, 36) = 5.523, *P* = 0.024] showing that the participants were hungrier at the end than at the beginning of the sessions. No other significant interactions were noted.

#### Liking and Wanting as Functions of Metabolic State

The mean liking and wanting scores were calculated in the hunger and satiety states for food and Nfood odors for the three groups. MANOVA mainly indicated significant main effects for task and group as well for food [Wilks’ *λ*(13, 132) = 2.164, *P =* 0.014 and Wilks’ *λ*(13, 133) = 11.755, *P* < 0.001, respectively) as Nfood odors [Wilks’ λ(13, 132) = 13.809, *P <* 0.001, and Wilks’ *λ*(13, 133) = 1.804, *P* = 0.011, respectively], suggesting that the subjects in the three groups might have used different cognitive processes during the food and Nfood liking and wanting tasks.

**FIGURE 3:**
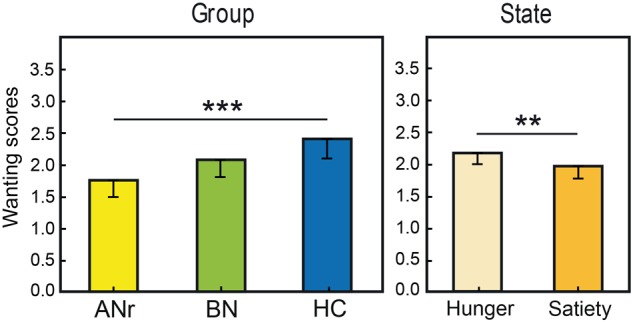
Mean rating scores reported for food odors in the wanting task as a function of group and metabolic state. Vertical bars, s.e.m.; ^∗∗^*P* < 0.01; ^∗∗∗^*P* < 0.001.

We performed separate three-way (state × odor × group) ANOVAs for liking and wanting data. For food odors delivered in the liking task, ANOVA revealed a significant odor x group interaction [*F*(26, 468) = 1.658, *P* = 0.023], but the differences between the groups were not significant for any single odor (*P* > 0.05). For Nfood odors delivered in the liking task, no significant effect was observed (*P* > 0.05). For food odors delivered in the wanting task, we found significant effects of group [*F*(2, 36) = 5.401, *P* = 0.009] and state [*F*(1, 36) = 7.524, *P* = 0.009] factors (Figure 3). The group effect was due to ANr participants reporting lower scores than HC participants (*P* = 0.002, α_adjusted_ = 0.017). When considering the three groups together, the wanting scores were significantly higher in the hunger state than in the satiety state.

We then distinguished between odors evoking high EDF (bacon, beef, blue cheese, Gruyère cheese, pâté, peanut, pizza, smoked bacon) and low EDF (apricot, banana, bitter almond, potato, shellfish, strawberry). For the odors evoking high EDF in the liking task, three-way (state × odor × group) ANOVA showed only a significant group effect [*F*(2, 36) = 3.169, *P* = 0.054], mainly due to the significantly lower rating scores of ANr participants compared with HC participants (*P* = 0.017 α_adjusted_ = 0.017), and a significant state effect [*F*(1, 36) = 6.049, *P* = 0.019] indicating a higher liking score in the hunger state than in the satiety state for all three groups. The analysis of wanting scores for odors evoking high EDF revealed a strong group effect [*F*(2, 36) = 14.80, *P* < 0. 001] with lower rating scores for ANr and BN patients than for HC participants (*P’s* < 0.001, α_adjusted_ = 0.017). No significant effect was observed for odors evoking low EDF (*P* > 0.05). These results indicated that differences in rating scores among groups were mainly observed for high EDF odors. We further observed a state effect [*F*(1, 36) = 15.801, *P* < 0.001] due to significantly higher rating scores reported during the hunger state than during the satiety state for all three groups.

#### Identification Performance and Response Times

We assessed the identification performances during the wanting task as a function of group and metabolic state using signal-detection theory [Hunger (HC: *d*’*_L_* = 2.823 ± 0.799, *C_L_* = -0.199 ± 0.436; ANr: *d*’*_L_* = 2.776 ± 1.340; *C_L_* = -0.094 ± 1.089; BN: *d*’*_L_* = 2.604 ± 1.038; *C_L_* = 0.060 ± 0.726); Satiety (HC: *d*’*_L_* = 2.923 ± 0.834, *C_L_* = -0.210 ± 0.368; ANr: *d*’*_L_* = 2.067 ± 1.311; *C_L_* = 0.234 ± 0.766 BN: *d*’*_L_* = 2.821 ± 1.147; *C_L_* = -0.233 ± 0.860)]. Two-way (state × group) ANOVAs revealed no significant main effects or interactions for the discrimination measure (*d*’*_L_*) [*F*(2, 36) ≤ 2.36, *P* ≥ 0.060] or for the bias responses (*C_L_*) [*F*(2, 36) ≤ 0.582, *P* ≥ 0.138]. These results indicate that ANr and BN patients correctly distinguished food and Nfood odors in the hunger and satiety states during the wanting task at the same rate as HC subjects and showed similar numbers of bias responses.

Response time (RT) was defined as the interval between odorant delivery and the subject’s response. ANOVAs did not reveal significant differences among groups for the liking or wanting tasks. First, for the liking task, a four-way (group × category × state × odor) ANOVA with repeated measurements on the last three factors mainly indicated a significant effect of category due to lower RTs for food than for Nfood odors [*F*(1, 36) = 52.57, *P* < 0.001], suggesting that as long as a food is not identified, it requires more time processing in olfactory memory to be sure that the odor does not evoke a Nfood. For the wanting task of food odors, a three-way (group × state × odor) ANOVA with repeated measurements on the last two factors did not revealed significant effects or interactions.

The participants were asked to breathe regularly and to avoid sniffing throughout the experiment, although variations in breathing amplitude were expected due to the experimental conditions and the participants’ answers during the rating tasks. As breathing variations are known to impact brain activation (Sobel et al., 1998), we analyzed the amplitude of the first inspiratory cycle following each odor stimulation and the first cycle preceding it. Mean cycle amplitudes were computed for the different experimental conditions. Four-way (group × state × category × inspiration) ANOVAs were performed for the two tasks, mainly revealing a significant category effect [liking: *F*(1, 36) = 8.444, *P* = 0.006; wanting: *F*(1, 36) = 4.295, *P* = 0.045] due to significantly lower inspiratory volumes for food than Nfood stimuli, but no significant difference in inspiratory volume was observed among groups [liking: *F*(2, 36) = 0.472, *P* = 0.628; wanting: *F*(2, 36) = 0.764, *P* = 0.473]. Thus, the differences in activation patterns among the groups could not be explained by differences in breathing.

### Cerebral Imaging Data

#### VOI Analyses

**FIGURE 4:**
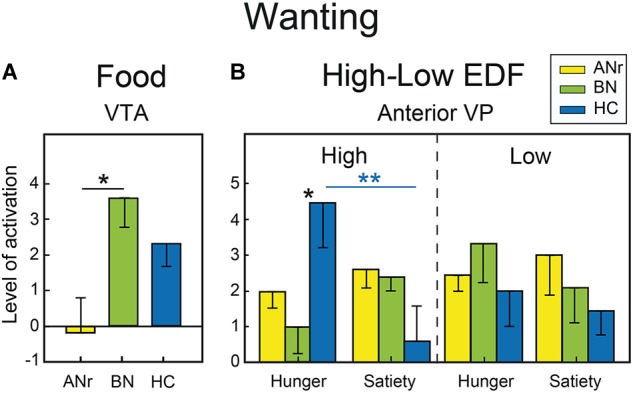
**(A)** Food odors: differential activation in the wanting task in the VTA as a function of group. **(B)** High vs. low EDF odors: differential activation in the wanting task in the anterior VP as a function of metabolic state and group. Vertical bars, s.e.m. ^∗^*P* < 0.05; ^∗∗^*P* < 0.01; ^∗∗∗^*P* < 0.001.

As the liking scores did not differ significantly among the three groups for food and Nfood odors, we did not examine the corresponding cerebral activation patterns. For food odors delivered during the wanting task, we compared the cerebral activation patterns among the three groups of participants as a function of hunger vs. satiety [(side × state × group) ANOVA)]. Anatomical VOI analyses revealed significant differences in the VTA (Figure 4A), and the anterior ventral pallidum (VP). For the VTA, we found a significant group effect [*F*(2, 36) = 3.975, *P* = 0.028]. Following correction for multiple comparisons, the T_max_ statistic indicated that activation was significantly lower in ANr than in BN patients (*P* = 0.047). For the anterior VP, we found a significant state x group interaction [*F*(2, 36) = 3.356, *P* = 0.046]. After correction for multiple comparisons (T_max_ statistic), only HC participants revealed an increased activation of VP in the hunger state than in the satiety state (*P* = 0.037). We also conducted VOI analyses in participants exposed to odors evoking high EDF because wanting scores differentiated the three groups of participants for this condition. However, no correction of alpha risk by the number of VOIs was needed because we were only interested to check that the same effects were found for high EDF odors as those previously obtained for food odors. A Bonferroni correction was applied for correcting the number of comparisons performed in a VOI. We examined the activation patterns by distinguishing those associated with high and low EDF odors [(side × state × category × group) ANOVA]. For the anterior VP, a significant state × category × group interaction [*F*(2, 36) = 3.242, *P* = 0.051] was detected, primarily due to higher activation in the hunger state than in the satiety state in HC participants for high EDF odors only (*P* = 0.0025, α_adjustedfor 0.01_ = 0.003) as depicted in Figure 4B. Activation in the hunger state for high EDF odors was also greater in HC than in BN (*P* = 0.006, α_adjusted_ = 0.017) participants, the difference between HC and ANr participants not reaching the level of significance (*P* = 0.040, α_adjusted_ = 0.017).

#### Whole-Brain Analyses

**FIGURE 5:**
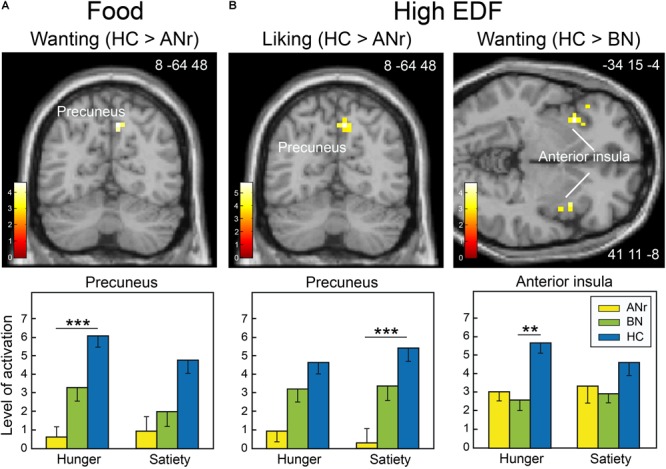
Brain sections showing differentially activated regions among the participants of the three groups when they were stimulated **(A)** with food odors in the hunger state in the wanting task and **(B)** with high EDF odors in the liking and wanting tasks in the satiety and hunger states, respectively. Color scales indicate *T*-values. Bar graphs show the mean levels of activation in response to food odors in the three groups. The levels of activation given for a metabolic state but not indicated with an asterisk are given for comparison. Vertical bars, s.e.m. ^∗∗^*P* < 0.01; ^∗∗∗^*P* < 0.001.

We primarily observed activation differences in the precuneus in ANr patients ([HC – ANr] contrast) stimulated with food odors in the wanting task (hunger state: xyz = 8 -64 48; *T* = 4.58; *k* = 11) and in the liking task (hunger: xyz = 11 -64 40; *T* = 4.44; *k* = 6; satiety: xyz = 11 -64 48; *T* = 6.45; *k* = 15). We further examined the activation patterns associated with high EDF odors in the two tasks. The [HC – ANr] contrast revealed mainly reduced activation for the precuneus in ANr patients in the satiety state in the liking task (xyz = 8 -64 48; *T* = 4.84; *k* = 15, Figure 5). In the wanting task, we further observed reduced bilateral activation of the anterior insula in BN patients in the hunger state (xyz = 41 11 -8; *T* = 3.63; *k* = 14, Figure 5).

**FIGURE 6:**
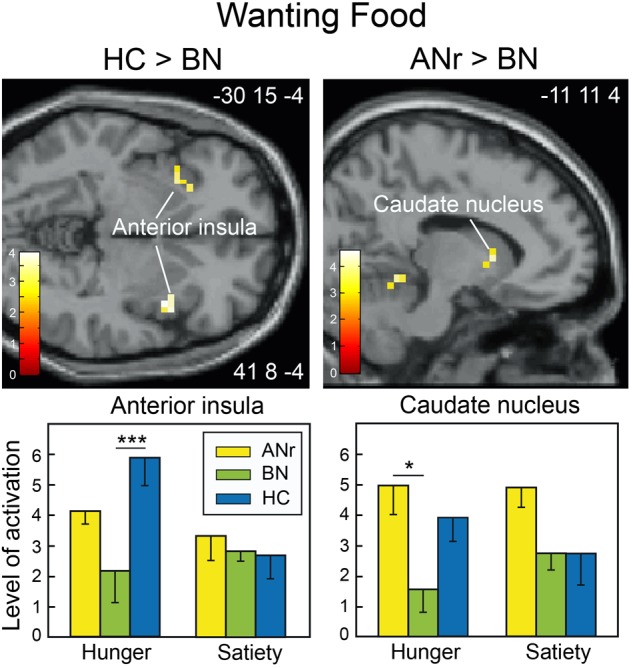
Brain sections showing differentially activated regions among participants of the three groups when stimulated with odors identified by participants as food odors in the wanting task during the hunger state. Color scales indicate *T*-values. Bar graphs show the mean levels of activation to food odors in the three groups. The levels of activation during the satiety state were extracted from the same clusters as found during the hunger state and are shown for comparison. Vertical bars, s.e.m. ^∗^*P* < 0.05; ^∗∗∗^*P* < 0.001.

Because the participants had to decide whether odors evoked food or Nfood before giving a wanting rating score, we used their responses to distinguish between the two odor categories rather than using the selection established *a priori* by the experimenters (Table 2). We then compared the cerebral activation patterns among the three groups as a function of the two metabolic states. As in previous analyses, we again found reduced activation of the precuneus in ANr patients as compared to HC participants ([HC – ANr] contrast: xyz = 8 -68 48; *T* = 4.44; *k* = 11). BN patients also exhibited lower activation of the anterior insula in the hunger state than did HC participants ([HC – BN] contrast: xyz = 41 8 -4; *T* = 3.78; *k* = 17), corroborating the results described above. For food wanting, we further found significantly lower activation of the caudate nucleus in the hunger state in BN patients than in ANr or HC participants (Figure 6, xyz = -15 11 8; *T* = 3.93; *k* = 6).

## Discussion

We investigated the behavioral and neural correlates of the subjective experiences of liking and wanting for food odors in women with two forms of EDs. ANr patients liked fewer odors evoking high EDF and wanted to eat odor-cued food less than did HC participants (regardless of energy density), and this food wanting difference was present to a lesser degree in BN patients for high EDF odors. ANr patients exhibited lower activation of the VTA than BN patients when evaluating the desire to eat odor-cued food. BN patients revealed a lack of activation of the caudate nucleus to food odors during the wanting task. In the two groups, a low desire to eat food was concomitant with reduced activation of the anterior VP and the anterior insula during the hunger state when presented high EDF odors. Finally, ANr patients exhibited lower activation of the precuneus than HC participants for several experimental conditions.

### Behavioral Data

Here, the smelling of high EDF odors revealed that ANr patients liked these food odors less than did HC participants (regardless of the metabolic state), while the liking scores of BN patients were between those of the other two groups but not significantly different from either. Thus, ANr patients, but not BN patients, displayed clearly decreased sensory pleasure in response to odors evoking high EDF. These results are consistent with studies showing (i) the influence of macronutrient composition or caloric load of food stimuli on self-reported pleasure in ANr patients (Giel et al., 2010; Jiang et al., 2010), (ii) an increased aversion to fat in AN compared with their BN counterparts (Sunday and Halmi, 1990), and (iii) a lower pleasantness ratings to high concentrations of sucrose in bulimic patients with a history of ANr compared with those without a history of AN (Franko et al., 1994).

When we examined the desire to eat food, ANr patients revealed lower wanting scores than HC participants for the 14 food odors used, whereas BN reported lower wanting scores than HC participants only for odors evoking high EDF. Thus, the desire to eat odor-cued food was influenced by the food nutritional content in both groups of patients although less influenced in BN than in ANr patients. These findings are in line with a previous study showing that ANr patients reported lower desire to eat palatable food evoked by pictures than HC participants during hunger state suggesting their failure to activate the appetitive motivational system (Soussignan et al., 2010).

The lower self-reported reward scores for food odors in ANr and BN patients than in HC participants raise the question of whether the patients’ olfactory abilities were impaired. Olfactory performance has been tested previously, although the reported findings are contradictory (Fedoroff et al., 1995; Aschenbrenner et al., 2008; Rapps et al., 2010; Schecklmann et al., 2012; Dazzi et al., 2013). Islam et al. (2015) emphasized that conflicting results could be due to differences in methodology. However, here, we noted higher suprathreshold detection scores in patients than in HC participants, indicating that the patients correctly detected odors. Further, we found different liking and wanting scores among the three groups only for high EDF odors, suggesting that the patients were able to identify different food odors, with lower scores thus reflecting hyporesponsivity to food reward cues.

### Functional Neuroimaging Data

We showed that the neural correlates of subjective experiences of pleasure and desire for food odors partly differed among the three groups.

#### Deactivation of VTA in ANr

ANr patients failed to activate the VTA compared with the two other groups during the wanting task. Evidence from neuroimaging studies has indicated that the VTA preferentially responds to expectation or delivery of primary (e.g., taste) or secondary (e.g., monetary) rewards but not to the delivery/anticipation of nonrewarding stimuli (O’Doherty et al., 2002; D’Ardenne et al., 2008). These findings are consistent with animal studies suggesting the involvement of VTA in the incentive salience attribution to neural representations of rewarding stimuli (Kelley and Berridge, 2002). For example, selective dopaminergic (DA) lesions of the VTA in rodents impair the consumption or the preference of taste stimuli (Shimura et al., 2002). The administration of orexins into the VTA induces reward-seeking behavior (Harris et al., 2005), whereas that of baclofen blocks both the firing of nucleus accumbens (NAc) neurons and the behavioral response to a cue predicting sucrose reward (Yun et al., 2004). Finally, optogenetic excitation of midbrain GABA neurons that inhibit VTA DA neurons elicits aversion (Tan et al., 2012). Here, the lack of activation of the VTA in ANr patients in response to food odors perceived as aversive to them is consistent with the idea that the assignment of incentive salience to food odors via the mesolimbic pathway is altered in them.

#### A Least Activation of Anterior VP and Insula in ANr and BN

ANr and BN patients (relative to HC participants) smelling high EDF odors in the hunger state revealed low activation of the anterior VP and the anterior insula during the wanting task. This result for the VP is reminiscent of our recent study revealing a lack of activation in this area in subjects disgusted by cheese and stimulated by cheese odors and images (Royet et al., 2016). The VP is considered an essential convergent point for hedonic and motivational signaling pathways in the brain (Smith et al., 2009). It receives DA inputs from VTA (Smith and Kieval, 2000) and GABAergic inputs from NAc (Pierce and Kumaresan, 2006). Its motivation-related activation is observed when food odors predict immediate arrival of their associated drink (Small et al., 2008) and when high-calorie food images are presented to obese women (Stoeckel et al., 2008). It is also linearly related to the amount of physical force produced in order to earn larger monetary rewards (Pessiglione et al., 2007). Thus, a VP reduced activation in the hunger state for odors during food wanting in ANr and BN patients suggests a possible dysregulation of this brain area which might impair the attribution of motivational salience and the associated experience of desire in these patients.

The anterior insula is an area of multisensory integration that responds to taste and olfactory stimuli (Small et al., 2004; Veldhuizen et al., 2010; Mazzola et al., 2017) and plays key roles in flavor perception and feeding behavior. Neuroimaging studies have shown greater activation to food-related visual stimuli during hunger in healthy subjects (LaBar et al., 2001; Malik et al., 2011) but reduced activation to food cues in the satiety state as the reward value of stimuli decreased (Small et al., 2001). In patients who did or did not recover from AN or BN, several studies have revealed reduced activation of the anterior insula in response to rewarding stimuli (food images, taste of sucrose) (Wagner et al., 2008; Bohon and Stice, 2011; Oberndorfer T.A. et al., 2013). These data are consistent with the present findings and the indication that the appetitive properties of food stimuli are likely lost in ANr and BN patients.

#### Deactivation of Caudate Nucleus in BN

Compared with HC participants, BN patients showed reduced activation of the caudate nucleus in the hunger state during the wanting task when exposed to odors evoking food. Previous studies in BN patients have revealed structural and functional alterations in the dorsal striatum. For example, compared with control women, BN patients showed reduced gray matter volume in the caudate nucleus (Coutinho et al., 2015), decreased dopamine D2 receptor binding in the posterior striatum (Broft et al., 2012), and reduced activation of the dorsal striatum associated with impaired behavioral inhibition (Skunde et al., 2016). The dorsal striatal DA pathway is involved in motivation processes related to reward processing and in executive functions supporting decision-making and reward-based action selection/reinforcement (Everitt and Robbins, 2005; Balleine et al., 2007). A hypodopaminergic striatal state may also contribute to impaired reward-related learning and may affect the ability of patients to utilize alternative reinforcers to binge eating and/or purging (Broft et al., 2012). Our findings are consistent with these studies, suggesting that the blunted activation of the dorsal striatal regions to food odors in BN patients might reflect an alteration of reward-related executive functions (e.g., decision making, selection of reward-based action and motor control), which may contribute to binge-eating and purging behaviors (Skunde et al., 2016).

#### Deactivation of Precuneus in ANr

We found a lack of activation of the right precuneus for several experimental conditions in ANr patients. Cavanna and Trimble (2006) described a wide variety of potential functions of the precuneus, including visuo-spatial imagery, episodic memory retrieval and self-processing operations. They hypothesized a unifying function of this structure in self-mental representation and modulation of conscious processes. However, the precuneus may also be specifically related to awareness of one’s body, with precuneus deactivation reflecting body image distortion. For example, comparing differential activation between self and non-self images, Sachdev et al. (2008) reported that AN patients exhibit less activation of the precuneus than HC participants. This differential processing of self-images was the characteristic disturbance in this group and might explain why AN patients had a distorted view of body image. McAdams and Krawczyk (2014) also found a deactivation of the precuneus in a self-knowledge condition (“I am,” “I look”), but not in a perspective-taking condition (“I believe, friend believes”) in women recovering from AN. Moreover, the distortion of body image is further considered as a multidimensional symptom involving perceptive, affective, and cognitive dimensions (Cash and Deagle, 1997), with the perceptive component mainly related to alterations of the precuneus and the inferior parietal lobe (Gaudio and Quattrocchi, 2012).

#### Limitations of the Study

We acknowledged a number of limitations in the present study. First, it is important to underline that the sample size of groups was small and enrolled only women (although women have EDs at a higher rate than men), limiting the strength of our fMRI data. Second, we examined only neural correlates of subjective/conscious experiences of wanting and liking for food odors. Thus, it remains to investigate whether implicit components of motivational and affective processing are also differentially altered in ANr and BN patients when exposed to food odors because animal research on brain reward has shown that objective components of “liking” and “wanting” are key elements of food reward. Third, in our wanting task, the participants had to decide whether an odor evoked food or Nfood, and then to rate a wanting score if their response was positive. This procedure may be confusing because this task involved cognitive processing of the type of odor (food vs. non-food stimuli), motor control (response initiation or inhibition based on whether the odor is a food or not), and rating of wanting score if the odor evoked a food. However, our main objective was to compare performances or activation patterns between groups, and not between the two tasks. Therefore, behavioral and functional differences observed between groups of participants can be considered as valid.

## Conclusion

Our study provided evidence that food odors are relevant sensory probes to gain better insight into the dysfunction of the mesolimbic and striatal circuitry involved, among other functions, in food reward processing in patients with EDs. While ANr and BN patients shared similar hypoactivation of brain regions underlying, inter alia, emotion/reward processes (anterior VP and insula), the ANr patients revealed reduced activation of brain regions involved in particular in the attribution of incentive salience to food odors (VTA) and in self-referential/body image processing (precuneus). BN patients revealed more specific reactivity to food/high EDF odors with hypoactivation of the dorsal striatum in the hunger state, possibly reflecting dysfunction in reward-related executive functions. Additional studies are needed to elucidate the detailed mechanisms of dysfunction of the brain reward circuitry in these two EDs.

## Data Availability

All datasets generated for this study are included in the manuscript and/or the supplementary files.

## Ethics Statement

The study was conducted in accordance with the recommendations of the local Institutional Review Board according to French regulations on biomedical experiments with healthy volunteers (Ethical Research Committee of CPP-Sud Est II (n° CPP A07-03), DGS2007-0054, February 23, 2007). The protocole was approved by the Ethical Research Committee of CPP-Sud Est II. All subjects gave written informed consent in accordance with the Declaration of Helsinki.

## Author Contributions

TJ and J-PR conceptualized and designed the study, collected the data, and performed the statistical analyses. EC screened participants for neurological disorders. J-PR, RS, and TJ wrote the main manuscript text. All authors reviewed the final version of the manuscript.

## Conflict of Interest Statement

The authors declare that the research was conducted in the absence of any commercial or financial relationships that could be construed as a potential conflict of interest.

## Footnotes

1 https://www.nitrc.org/projects/mricron

## References

[B1] Aharoni R. Hertz M. M. (2012). Disgust sensitivity and anorexia nervosa. *Eur. Eat. Dis. Rev.* 20 106–110. 10.1002/erv.1124 21789779

[B2] American Psychiatric Association (1994). *Diagnostic and Statistical Manual of Mental Health Disorders* 4th edn. Washington DC: American Psychiatric Publishing 539–550.

[B3] American Psychiatric Association (2013). *Diagnostic and Statistical Manual of Mental Disorders* 5th edn. Arlington, VA: American Psychiatric Publishing 338–350.

[B4] Anderluh M. B. Tchanturia K. Rabe-Hesketh S. Treasure J. (2003). Childhood obsessive-compulsive personality traits in adult women with eating disorders: defining a broader eating disorder phenotype. *Am. J. Psychiatry* 160 242–247. 10.1176/appi.ajp.160.2.242 12562569

[B5] Aschenbrenner K. Scholze N. Joraschky P. Hummel T. (2008). Gustatory and olfactory sensitivity in patients with anorexia and bulimia in the course of treatment. *J. Psychiatry Res.* 43 129–137. 10.1016/j.jpsychires.2008.03.003 18423668

[B6] Bagby R. M. Parker J. D. Taylor G. J. (1994). The twenty-item Toronto Alexithymia Scale–I. Item selection and cross-validation of the factor structure. *J. Psychosom. Res.* 38 23–32. 10.1016/0022-3999(94)90005-1 8126686

[B7] Balleine B. W. Delgado M. R. Hikosaka O. (2007). The role of the dorsal striatum in reward and decision-making. *J. Neurosci.* 27 8161–8165. 10.1523/JNEUROSCI.1554-07.2007 17670959 PMC6673072

[B8] Banks W. P. (1970). Signal detection theory and human memory. *Psychol. Bull.* 74 81–99. 10.1037/h0029531

[B9] Beck A. T. Beamesderfer A. (1974). Assessment of depression: the depression inventory. *Mod. Prob. Pharmacopsychiatry* 7 151–169. 10.1159/000395074 4412100

[B10] Berridge K. C. (2009). ‘Liking’ and ‘wanting’ food rewards: brain substrates and roles in eating disorders. *Physiol. Behav.* 97 537–550. 10.1016/j.physbeh.2009.02.044 19336238 PMC2717031

[B11] Berridge K. C. Robinson T. E. (2016). Liking, wanting, and the incentive-sensitization theory of addiction. *Am. Psychol.* 71 670–679. 10.1037/amp0000059 27977239 PMC5171207

[B12] Bohon C. Stice E. (2011). Reward abnormalities among women with full and subthreshold bulimia nervosa: a functional magnetic resonance imaging study. *Int. J. Eat. Disord.* 44 585–595. 10.1002/eat.20869 21997421 PMC3111910

[B13] Broft A. I. Berner L. A. Martinez D. Walsh B. T. (2011). Bulimia nervosa and evidence for striatal dopamine dysregulation: a conceptual review. *Physiol. Behav.* 104 122–127. 10.1016/j.physbeh.2011.04.028 21549135 PMC3111921

[B14] Broft A. I. Shingleton R. Kaufman J. Liu F. Kumar D. Slifstein M. (2012). Striatal dopamine in bulimia nervosa: a PET imaging study. *Int. J. Eat. Disord.* 45 648–656. 10.1002/eat.20984 22331810 PMC3640453

[B15] Cash T. F. Deagle E. A. III (1997). The nature and extent of body-image disturbances in anorexia nervosa and bulimia nervosa: a meta-analysis. *Int. J.f Eat. Disord.* 22 107–125. 10.1002/(SICI)1098-108X(199709)22:2<107::AID-EAT1>3.0.CO;2-J 9261648

[B16] Cavanna A. E. Trimble M. R. (2006). The precuneus: a review of its functional anatomy and behavioural correlates. *Brain* 129 564–583. 10.1093/brain/awl004 16399806

[B17] Chapman L. J. Chapman J. P. Raulin M. L. (1976). Scales for physical and social anhedonia. *J. Abn. Psychol.* 85 374–382. 10.1037/0021-843X.85.4.374 956504

[B18] Chen C. Witte M. Heemsbergen W. Van Herk M. (2013). Multiple comparisons permutation test for image based data mining in radiotherapy. *Radiat. Oncol.* 8:293. 10.1186/1748-717X-8-293 24365155 PMC3880053

[B19] Connan F. Campbell I. C. Katzman M. Lightman S. L. Treasure J. (2003). A neurodevelopmental model for anorexia nervosa. *Physiol. Behav.* 79 13–24. 10.1016/S0031-9384(03)00101-X 12818706

[B20] Corwin J. (1989). Olfactory identification in hemodialysis: acute and chronic effects on discrimination and response bias. *Neuropsychologia* 27 513–522. 10.1016/0028-3932(89)90056-0 2733824

[B21] Coutinho J. Ramos A. F. Maia L. Castro L. Conceicao E. Geliebter A. (2015). Volumetric alterations in the nucleus accumbens and caudate nucleus in bulimia nervosa: a structural magnetic resonance imaging study. *Int. J. Eat. Disord.* 48 206–214. 10.1002/eat.22273 24634102

[B22] Cowdrey F. A. Finlayson G. Park R. J. (2013). Liking compared with wanting for high- and low-calorie foods in anorexia nervosa: aberrant food reward even after weight restoration. *Am. J. Clin. Nut.* 97 463–470. 10.3945/ajcn.112.046011 23364019

[B23] Cowdrey F. A. Park R. J. Harmer C. J. Mccabe C. (2011). Increased neural processing of rewarding and aversive food stimuli in recovered anorexia nervosa. *Biol Psychiatry* 70 736–743. 10.1016/j.biopsych.2011.05.028 21714958

[B24] D’Ardenne K. Mcclure S. M. Nystrom L. E. Cohen J. D. (2008). BOLD responses reflecting dopaminergic signals in the human ventral tegmental area. *Science* 319 1264–1267. 10.1126/science.1150605 18309087

[B25] Dazzi F. Nitto S. D. Zambetti G. Loriedo C. Ciofalo A. (2013). Alterations of the olfactory-gustatory functions in patients with eating disorders. *Eur. Eat. Dis. Rev.* 21 382–385. 10.1002/erv.2238 23788398

[B26] Diekhof E. K. Kaps L. Falkai P. Gruber O. (2012). The role of the human ventral striatum and the medial orbitofrontal cortex in the representation of reward magnitude - an activation likelihood estimation meta-analysis of neuroimaging studies of passive reward expectancy and outcome processing. *Neuropsychologia* 50 1252–1266. 10.1016/j.neuropsychologia.2012.02.007 22366111

[B27] Duvernoy H. M. (1999). *The Human Brain Surface, Three-Dimensional Sectional Anatomy with MRI, and Blood Supply.* New York, NY: Springer.

[B28] Everitt B. J. Robbins T. W. (2005). Neural systems of reinforcement for drug addiction: from actions to habits to compulsion. *Nat. Neurosci.* 8 1481–1489. 10.1038/nn1579 16251991

[B29] Fedoroff I. C. Stoner S. A. Andersen A. E. Doty R. L. Rolls B. J. (1995). Olfactory dysfunction in anorexia and bulimia nervosa. *Int. J. Eat. Disord.* 18 71–77. 10.1002/1098-108X(199507)18:1<71::AID-EAT2260180108>3.0.CO;2-5 7670445

[B30] Finlayson G. Arlotti A. Dalton M. King N. Blundell J. E. (2011). Implicit wanting and explicit liking are markers for trait binge eating. A susceptible phenotype for overeating. *Appetite* 57 722–728. 10.1016/j.appet.2011.08.012 21896296

[B31] Frank G. K. Reynolds J. R. Shott M. E. Jappe L. Yang T. T. Tregellas J. R. (2012). Anorexia nervosa and obesity are associated with opposite brain reward response. *Neuropsychopharmacology* 37 2031–2046. 10.1038/npp.2012.51 22549118 PMC3398719

[B32] Franko D. L. Wolfe B. E. Jimerson D. C. (1994). Elevated sweet taste pleasantness ratings in bulimia nervosa. *Physiol. Behav.* 56 969–973. 10.1016/0031-9384(94)90331-X 7824599

[B33] Friederich H. C. Wu M. Simon J. J. Herzog W. (2013). Neurocircuit function in eating disorders. *Int. J. Eat. Disord.* 46 425–432. 10.1002/eat.22099 23658085

[B34] Friston K. J. Ashburner J. Frith C. Poline J. B. Healther J. D. Frackowiak R. S. (1995a). Spatial registration and normalization of images. *Hum. Brain Mapp.* 2 165–189. 10.1002/hbm.460030303

[B35] Friston K. J. Holmes A. P. Worsley K. J. Poline J. B. Frith C. Frackowiak R. S. (1995b). Statistical parametric maps in functional imaging: a general linear approach. *Hum. Brain Mapp.* 2 189–210. 10.1002/hbm.460020402

[B36] Friston K. J. Josephs O. Rees G. Turner R. (1998). Nonlinear event-related responses in fMRI. *Magn. Res. Med.* 39 41–52. 10.1002/mrm.1910390109 9438436

[B37] Gaudio S. Quattrocchi C. C. (2012). Neural basis of a multidimensional model of body image distortion in anorexia nervosa. *Neurosci. Biobehav. Rev.* 36 1839–1847. 10.1016/j.neubiorev.2012.05.003 22613629

[B38] Giel K. E. Teufel M. Friederich H. C. Hautzinger M. Enck P. Zipfel S. (2010). Processing of pictorial food stimuli in patients with eating disorders - a systematic review. *Int. J. Eat. Disord.* 44 105–117. 10.1002/eat.20785 20127931

[B39] Gizewski E. R. Rosenberger C. De Greiff A. Moll A. Senf W. Wanke I. (2010). Influence of satiety and subjective valence rating on cerebral activation patterns in response to visual stimulation with high-calorie stimuli among restrictive anorectic and control women. *Neuropsychobiology* 62 182–192. 10.1159/000319360 20664231

[B40] Handwerker D. A. Ollinger J. M. D’esposito M. (2004). Variation of BOLD hemodynamic responses across subjects and brain regions and their effects on statistical analyses. *Neuroimage* 21 1639–1651. 10.1016/j.neuroimage.2003.11.029 15050587

[B41] Harris G. C. Wimmer M. Aston-Jones G. (2005). A role for lateral hypothalamic orexin neurons in reward seeking. *Nature* 437 556–559. 10.1038/nature04071 16100511

[B42] Harrison A. O’brien N. Lopez C. Treasure J. (2010). Sensitivity to reward and punishment in eating disorders. *Psychiatry Res.* 177 1–11. 10.1016/j.psychres.2009.06.010 20381877

[B43] Henderson M. Freeman C. P. (1987). A self-rating scale for bulimia. The ’BITE’. *Br. J. Psychiatry* 150 18–24. 10.1192/bjp.150.1.18 3651670

[B44] Holsen L. M. Lawson E. A. Blum J. Ko E. Makris N. Fazeli P. K. (2012). Food motivation circuitry hypoactivation related to hedonic and nonhedonic aspects of hunger and satiety in women with active anorexia nervosa and weight-restored women with anorexia nervosa. *J. Psychiatry Neurosci.* 37 322–332. 10.1503/jpn.110156 22498079 PMC3447131

[B45] Hopfinger J. B. Buchel C. Holmes A. P. Friston K. J. (2000). A study of analysis parameters that influence the sensitivity of event-related fMRI analyses. *Neuroimage* 11 326–333. 10.1006/nimg.2000.0549 10725188

[B46] Islam M. A. Fagundo A. B. Arcelus J. Aguera Z. Jimenez-Murcia S. Fernandez-Real J. M. (2015). Olfaction in eating disorders and abnormal eating behavior: a systematic review. *Front. Psychol.* 6:1431. 10.3389/fpsyg.2015.01431 26483708 PMC4588114

[B47] Jansen A. (1998). A learning model of binge eating: cue reactivity and cue exposure. *Behav. Res. Ther.* 36 257–272. 10.1016/S0005-7967(98)00055-2 9642846

[B48] Jiang T. Schaal B. Boulanger V. Kontar F. Soussignan R. (2013). Children’s reward responses to picture- and odor-cued food stimuli: a developmental analysis between 6 and 11years. *Appetite* 67 88–98. 10.1016/j.appet.2013.04.003 23583313

[B49] Jiang T. Soussignan R. Rigaud D. Martin S. Royet J. P. Brondel L. (2008). Alliesthesia to food cues: heterogeneity across stimuli and sensory modalities. *Physiol. Behav.* 95 464–470. 10.1016/j.physbeh.2008.07.014 18675834

[B50] Jiang T. Soussignan R. Rigaud D. Schaal B. (2010). Pleasure for visual and olfactory stimuli evoking energy-dense foods is decreased in anorexia nervosa. *Psychiatry Res.* 180 42–47. 10.1016/j.psychres.2010.04.041 20488559

[B51] Jiang T. Soussignan R. Schaal B. Royet J. P. (2015). Reward for food odors: an fMRI study of liking and wanting as a function of metabolic state and BMI. *Soc. Cogn. Affect. Neurosci.* 10 561–568. 10.1093/scan/nsu086 24948157 PMC4381239

[B52] Katsyri J. Hari R. Ravaja N. Nummenmaa L. (2013). Just watching the game ain’t enough: striatal fMRI reward responses to successes and failures in a video game during active and vicarious playing. *Front. Hum. Neurosci.* 7:278. 10.3389/fnhum.2013.00278 23781195 PMC3680713

[B53] Kaye W. H. Bulik C. M. Thornton L. Barbarich N. Masters K. (2004). Comorbidity of anxiety disorders with anorexia and bulimia nervosa. *Am. J. Psychiatry* 161 2215–2221. 10.1176/appi.ajp.161.12.2215 15569892

[B54] Kaye W. H. Wierenga C. E. Bailer U. F. Simmons A. N. Wagner A. Bischoff-Grethe A. (2013). Does a shared neurobiology for foods and drugs of abuse contribute to extremes of food ingestion in anorexia and bulimia nervosa? *Biol. Psychiatry* 73 836–842. 10.1016/j.biopsych.2013.01.002 23380716 PMC3755487

[B55] Kelley A. E. Berridge K. C. (2002). The neuroscience of natural rewards: relevance to addictive drugs. *J. Neurosci.* 22 3306–3311. 10.1523/JNEUROSCI.22-09-03306.2002 11978804 PMC6758373

[B56] Kennedy S. H. Kaplan A. S. Garfinkel P. E. Rockert W. Toner B. Abbey S. E. (1994). Depression in anorexia nervosa and bulimia nervosa: discriminating depressive symptoms and episodes. *J. Psychosom. Res.* 38 773–782. 10.1016/0022-3999(94)90030-2 7877132

[B57] Kühn S. Gallinat J. (2012). The neural correlates of subjective pleasantness. *Neuroimage* 61 289–294. 10.1016/j.neuroimage.2012.02.065 22406357

[B58] LaBar K. S. Gitelman D. R. Parrish T. B. Kim Y. H. Nobre A. C. Mesulam M. M. (2001). Hunger selectively modulates corticolimbic activation to food stimuli in humans. *Behav. Neurosci.* 115 493–500. 10.1037/0735-7044.115.2.493 11345973

[B59] Liu X. Hairston J. Schrier M. Fan J. (2011). Common and distinct networks underlying reward valence and processing stages: a meta-analysis of functional neuroimaging studies. *Neurosci. Biobehav. Rev.* 35 1219–1236. 10.1016/j.neubiorev.2010.12.012 21185861 PMC3395003

[B60] Lockhart R. S. Murdock B. B. (1970). Memory and the theory of signal detection. *Psychol. Bull.* 74 100–109. 10.1037/h0029536

[B61] Lule D. Schulze U. M. Bauer K. Scholl F. Muller S. Fladung A. K. (2014). Anorexia nervosa and its relation to depression, anxiety, alexithymia and emotional processing deficits. *Eat. Weight Disord.* 19 209–216. 10.1007/s40519-014-0101-z 24474662

[B62] Mai J. K. Paxinos G. Voss T. (2008). *Atlas of the Human Brain.* San Diego, CA: Academic Press.

[B63] Malik S. Mcglone F. Dagher A. (2011). State of expectancy modulates the neural response to visual food stimuli in humans. *Appetite* 56 302–309. 10.1016/j.appet.2011.01.005 21232571

[B64] Mawlawi O. Martinez D. Slifstein M. Broft A. Chatterjee R. Hwang D. R. (2001). Imaging human mesolimbic dopamine transmission with positron emission tomography: I. Accuracy and precision of D(2) receptor parameter measurements in ventral striatum. *J. Cereb. Blood Flow Metab.* 21 1034–10357. 10.1097/00004647-200109000-00002 11524609

[B65] Mazzola L. Royet J. P. Catenoix H. Montavont A. Isnard J. Mauguiere F. (2017). Gustatory and olfactory responses to stimulation of the human insula. *Ann. Neurol.* 82 360–370. 10.1002/ana.25010 28796326

[B66] McAdams C. J. Krawczyk D. C. (2014). Who am I? How do I look? Neural differences in self-identity in anorexia nervosa. *Soc. Cogn. Affect. Neurosci.* 9 12–21. 10.1093/scan/nss093 22956668 PMC3871723

[B67] Mikl M. Marecek R. Hlustik P. Pavlicova M. Drastich A. Chlebus P. (2008). Effects of spatial smoothing on fMRI group inferences. *Magn. Reson. Imaging* 26 490–503. 10.1016/j.mri.2007.08.006 18060720

[B68] Monteleone A. M. Castellini G. Volpe U. Ricca V. Lelli L. Monteleone P. (2017). Neuroendocrinology and brain imaging of reward in eating disorders: a possible key to the treatment of anorexia nervosa and bulimia nervosa. *Prog. Neuropsychopharmacol. Biol. Psychiatry* 80(Pt B) 132–142. 10.1016/j.pnpbp.2017.02.020 28259721

[B69] Nichols T. E. Holmes A. P. (2001). Nonparametric permutation tests for functional neuroimaging: a primer with examples. *Hum. Brain Map.* 15 1–25. 10.1002/hbm.1058 11747097 PMC6871862

[B70] Oberndorfer T. Simmons A. Mccurdy D. Strigo I. Matthews S. Yang T. (2013). Greater anterior insula activation during anticipation of food images in women recovered from anorexia nervosa versus controls. *Psychiatry Res.* 214 132–141. 10.1016/j.pscychresns.2013.06.010 23993362 PMC3880160

[B71] Oberndorfer T. A. Frank G. K. Simmons A. N. Wagner A. Mccurdy D. Fudge J. L. (2013). Altered insula response to sweet taste processing after recovery from anorexia and bulimia nervosa. *Am. J. Psychiatry* 170 1143–1151. 10.1176/appi.ajp.2013.11111745 23732817 PMC3971875

[B72] O’Doherty J. P. Deichmann R. Critchley H. D. Dolan R. J. (2002). Neural responses during anticipation of a primary taste reward. *Neuron* 33 815–826. 10.1016/S0896-6273(02)00603-7 11879657

[B73] Oldfield R. C. (1971). The assessment and analysis of handedness: the Edinburgh inventory. *Neuropsychologia* 9 97–113. 10.1016/0028-3932(71)90067-4 5146491

[B74] Park R. J. Godier L. R. Cowdrey F. A. (2014). Hungry for reward: How can neuroscience inform the development of treatment for Anorexia Nervosa? *Behav. Res. Ther.* 62 47–59. 10.1016/j.brat.2014.07.007 25151600

[B75] Pessiglione M. Schmidt L. Draganski B. Kalisch R. Lau H. Dolan R. J. (2007). How the brain translates money into force: a neuroimaging study of subliminal motivation. *Science* 316 904–906. 10.1126/science.1140459 17431137 PMC2631941

[B76] Pierce R. C. Kumaresan V. (2006). The mesolimbic dopamine system: the final common pathway for the reinforcing effect of drugs of abuse? *Neurosci. Biobehav. Rev.* 30 215–238. 10.1016/j.neubiorev.2005.04.016 16099045

[B77] Raffi A. R. Rondini M. Grandi S. Fava G. A. (2000). Life events and prodromal symptoms in bulimia nervosa. *Psychol. Med.* 30 727–731. 10.1017/S0033291799002019 10883727

[B78] Rapps N. Giel K. E. Sohngen E. Salini A. Enck P. Bischoff S. C. (2010). Olfactory deficits in patients with anorexia nervosa. *Eur. Eat. Disord. Rev.* 18 385–389. 10.1002/erv.1010 20821739

[B79] Royet J. P. Meunier D. Torquet N. Mouly A. M. Jiang T. (2016). The neural bases of disgust for cheese: an fMRI Study. *Front. Hum. Neurosci.* 10:511. 10.3389/fnhum.2016.00511 27799903 PMC5065955

[B80] Sachdev P. Mondraty N. Wen W. Gulliford K. (2008). Brains of anorexia nervosa patients process self-images differently from non-self-images: an fMRI study. *Neuropsychologia* 46 2161–2168. 10.1016/j.neuropsychologia.2008.02.031 18406432

[B81] Sanders N. Smeets P. A. Van Elburg A. A. Danner U. N. Van Meer F. Hoek H. W. (2015). Altered food-cue processing in chronically ill and recovered women with anorexia nervosa. *Front. Behav. Neurosci.* 9:46. 10.3389/fnbeh.2015.00046 25774128 PMC4342866

[B82] Schecklmann M. Pfannstiel C. Fallgatter A. J. Warnke A. Gerlach M. Romanos M. (2012). Olfaction in child and adolescent anorexia nervosa. *J. Neural Transm.* 119 721–728. 10.1007/s00702-011-0752-0 22183089

[B83] Shimura T. Kamada Y. Yamamoto T. (2002). Ventral tegmental lesions reduce overconsumption of normally preferred taste fluid in rats. *Behav. Brain Res.* 134 123–130. 10.1016/S0166-4328(01)00461-2 12191798

[B84] Skunde M. Walther S. Simon J. J. Wu M. Bendszus M. Herzog W. (2016). Neural signature of behavioural inhibition in women with bulimia nervosa. *J. Psychiatry Neurosci.* 41 E69–E78. 10.1503/jpn.150335 27575858 PMC5008924

[B85] Small D. M. Veldhuizen M. G. Felsted J. Mak Y. E. Mcglone F. (2008). Separable substrates for anticipatory and consummatory food chemosensation. *Neuron* 57 786–797. 10.1016/j.neuron.2008.01.021 18341997 PMC2669434

[B86] Small D. M. Voss J. Mak Y. E. Simmons K. B. Parrish T. Gitelman D. (2004). Experience-dependent neural integration of taste and smell in the human brain. *J. Neurophysiol.* 92 1892–1903. 10.1152/jn.00050.2004 15102894

[B87] Small D. M. Zatorre R. J. Dagher A. Evans A. C. Jones-Gotman M. (2001). Changes in brain activity related to eating chocolate: from pleasure to aversion. *Brain* 124 1720–1733. 10.1093/brain/124.9.1720 11522575

[B88] Smith K. S. Tindell A. J. Aldridge J. W. Berridge K. C. (2009). Ventral pallidum roles in reward and motivation. *Behav. Brain Res.* 196 155–167. 10.1016/j.bbr.2008.09.038 18955088 PMC2606924

[B89] Smith Y. Kieval J. Z. (2000). Anatomy of the dopamine system in the basal ganglia. *Trends Neurosci.* 23 S28–S33. 10.1016/S1471-1931(00)00023-9 11052217

[B90] Snodgrass J. G. Corwin J. (1988). Pragmatics of measuring recognition memory: applications to dementia and amnesia. *J. Exp. Psychol. Gen.* 117 34–50. 10.1037/0096-3445.117.1.34 2966230

[B91] Sobel N. Prabhakaran V. Desmond J. E. Glover G. H. Goode R. L. Sullivan E. V. (1998). Sniffing and smelling: separate subsystems in the human olfactory cortex. *Nature* 392 282–286. 10.1038/32654 9521322

[B92] Soussignan R. Jiang T. Rigaud D. Royet J. P. Schaal B. (2010). Subliminal fear priming potentiates negative facial reactions to food pictures in women with anorexia nervosa. *Psychol. Med.* 40 503–514. 10.1017/S0033291709990377 19619383

[B93] Soussignan R. Schaal B. Boulanger V. Gaillet M. Jiang T. (2012). Orofacial reactivity to the sight and smell of food stimuli. Evidence for anticipatory liking related to food reward cues in overweight children. *Appetite* 58 508–516. 10.1016/j.appet.2011.12.018 22245131

[B94] Soussignan R. Schaal B. Rigaud D. Royet J. P. Jiang T. (2011). Hedonic reactivity to visual and olfactory cues: rapid facial electromyographic reactions are altered in anorexia nervosa. *Biol. Psychol.* 86 265–272. 10.1016/j.biopsycho.2010.12.007 21185351

[B95] Stoeckel L. E. Weller R. E. Cook E. W. III Twieg D. B. Knowlton R. C. Cox J. E. (2008). Widespread reward-system activation in obese women in response to pictures of high-calorie foods. *Neuroimage* 41 636–647. 10.1016/j.neuroimage.2008.02.031 18413289

[B96] Sunday S. R. Halmi K. A. (1990). Taste perceptions and hedonics in eating disorders. *Physiol. Behav.* 48 587–594. 10.1016/0031-9384(90)90196-B 2082356

[B97] Tan K. R. Yvon C. Turiault M. Mirzabekov J. J. Doehner J. Labouebe G. (2012). GABA neurons of the VTA drive conditioned place aversion. *Neuron* 73 1173–1183. 10.1016/j.neuron.2012.02.015 22445344 PMC6690362

[B98] Uher R. Murphy T. Brammer M. J. Dalgleish T. Phillips M. L. Ng V. W. (2004). Medial prefrontal cortex activity associated with symptom provocation in eating disorders. *Am. J. Psychiatry* 161 1238–1246. 10.1176/appi.ajp.161.7.1238 15229057

[B99] Van Overveld W. J. M. De Jong P. J. Peters M. L. Cavanagh K. Davey G. C. L. (2006). Disgust and contamination sensitivity: separate constructs that are differentially related to specific fears. *Pers. Individ. Differ.* 41 1241–1252. 10.1016/j.paid.2006.04.021

[B100] Veldhuizen M. G. Nachtigal D. Teulings L. Gitelman D. R. Small D. M. (2010). The insular taste cortex contributes to odor quality coding. *Front. Hum. Neurosci.* 4:58. 10.3389/fnhum.2010.00058 20700500 PMC2917218

[B101] Vigouroux M. Bertrand B. Farget V. Plailly J. Royet J. P. (2005). A stimulation method using odors suitable for PET and fMRI studies with recording of physiological and behavioral signals. *J. Neurosci. Methods* 142 35–44. 10.1016/j.jneumeth.2004.07.010 15652615

[B102] Wagner A. Aizenstein H. Mazurkewicz L. Fudge J. Frank G. K. Putnam K. (2008). Altered insula response to taste stimuli in individuals recovered from restricting-type anorexia nervosa. *Neuropsychopharmacology* 33 513–523. 10.1038/sj.npp.1301443 17487228

[B103] Weygandt M. Schaefer A. Schienle A. Haynes J. D. (2012). Diagnosing different binge-eating disorders based on reward-related brain activation patterns. *Hum. Brain Mapp.* 33 2135–2146. 10.1002/hbm.21345 22887826 PMC6869845

[B104] Winer B. J. Brown D. R. Michels K. M. (1991). *Statistical Principles in Experimental Design.* New York, NY: McGraw-Hill.

[B105] Yun I. A. Wakabayashi K. T. Fields H. L. Nicola S. M. (2004). The ventral tegmental area is required for the behavioral and nucleus accumbens neuronal firing responses to incentive cues. *J. Neurosci.* 24 2923–2933. 10.1523/JNEUROSCI.5282-03.2004 15044531 PMC6729854

